# Targeted Screening Strategies for Head and Neck Cancer: A Global Review of Evidence, Technologies, and Cost-Effectiveness †

**DOI:** 10.3390/diagnostics15162095

**Published:** 2025-08-20

**Authors:** Orlando Guntinas-Lichius, Claudio Bücking, Sweet Ping Ng, Fernando López, Juan Pablo Rodrigo, Karthik N. Rao, Andrés Coca Pelaz, Luiz P. Kowalski, Cesare Piazza, Alessandra Rinaldo, Alfio Ferlito

**Affiliations:** 1 Department of Otorhinolaryngology, Jena University Hospital, Am Klinikum 1, D-07747 Jena, Germany; claudio.buecking@med.uni-jena.de; 2 Department of Radiation Oncology, Austin Health, Melbourne, VIC 3084, Australia; sweetping.ng@austin.org.au; 3 Department of Otolaryngology, Hospital Universitario Central de Asturias, University of Oviedo, Instituto de Investigación Sanitaria del Principado de Asturias (ISPA), The University Institute of Oncology of Asturias—Cajastur Social Programme (IUOPA), Centro de Investigación Biomédica en Red Cáncer (CIBERONC), 33011 Oviedo, Spain; fernandolopezphd@gmail.com (F.L.); jprodrigo@uniovi.es (J.P.R.); acocapelaz@yahoo.es (A.C.P.); 4 Department of Head and Neck Oncology, Sri Shankara Cancer Foundation, Bangalore 560004, India; karthik.nag.rao@gmail.com; 5 Head and Neck Surgery and Otorhinolaryngology Department, A C Camargo Cancer Center, Sao Paulo 01509-900, Brazil; lp_kowalski@uol.com.br; 6 Head and Neck Surgery, Faculty of Medicine, University of Sao Paulo, Sao Paulo 05508-020, Brazil; 7 Unit of Otorhinolaryngology—Head and Neck Surgery, ASST Spedali Civili of Brescia, Department of Surgical and Medical Specialties, Radiological Sciences, and Public Health, School of Medicine, University of Brescia, 25123 Brescia, Italy; cesare.piazza@unibs.it; 8 ENT Unit, Policlinico Città di Udine, 33100 Udine, Italy; dottalerinaldo@gmail.com; 9 Coordinator of the International Head and Neck Scientific Group, 35100 Padua, Italy; profalfioferlito@gmail.com

**Keywords:** head and neck cancer, cancer screening, opportunistic screening, early detection, high-risk populations, liquid biopsy, human papillomavirus (HPV), Epstein-Barr virus (EBV), cost-effectiveness, biophotonics

## Abstract

Head and neck cancer (HNC) is the seventh most common cancer worldwide, with rising incidence particularly in oropharyngeal cancer subsites. Despite well-known risk factors, such as tobacco and alcohol consumption as well as human papillomavirus (HPV) infection, most HNCs are diagnosed at an advanced stage, resulting in poor prognosis. Early detection and screening are critical, especially in high-risk populations. Nevertheless, there is a lack of guidelines for a stratified HNC screening. A systematic literature review was conducted following PRISMA guidelines, using PubMed and ScienceDirect databases up to 30 June 2025. Search terms included “screening”, “early diagnosis”, and specific HNC subsites. A total of 199 records were screened, and 160 studies were included based on relevance and scientific rigor. The review concentrates on contemporary screening modalities, stratification of high-risk cohorts, emerging technologies, and cost-effectiveness evidence. Visual inspection and panendoscopy remain the standard tools for HNC screening, but have limited effectiveness and cost-efficiency. Opportunistic screening in high-risk individuals, especially in regions with high HNC prevalence, has shown benefits. Liquid biopsy techniques targeting HPV- and Epstein-Barr virus-related HNC demonstrate high sensitivity for early detection and recurrence monitoring. Novel imaging technologies like narrow-band imaging and Raman spectroscopy show promising diagnostic accuracy but require further validation. Most broad-based screening programs lack cost-effectiveness, while targeted strategies in high-risk groups appear more viable. Screening for HNC should be stratified by individual risk profiles and regional disease prevalence. Emerging technologies, particularly liquid and optical biopsy techniques, offer transformative potential. Future screening strategies must integrate technological advances into tailored, evidence-based protocols to improve early detection and patient outcomes in HNC.

## 1. Introduction

Head and neck cancer (HNC) constitutes the world’s seventh most prevalent malignancy [1]. Oral cancer is the second most prevalent malignancy in India, and nasopharyngeal cancer ranks eleventh in China. Overall, HNC accounts for more than 660,000 new cases and 325,000 deaths annually [1]. Although there is a decline in smoking as an important risk factor, HNC incidence is increasing and is projected to continue to rise, largely driven by increases in the number of oropharyngeal cancers [2]. The major traditional risk factors of HNC are tobacco smoking in combination with alcohol consumption [3]. Smokeless tobacco and areca nut use are the main risk factors globally for oral cancer. These factors cause roughly one in three cases of oral cancer worldwide. Moreover, nearly 9 in 10 (88%) of all oral cancer cases in South-Central Asia, and more than 95% occurring in low- and middle-income countries, are associated with smokeless tobacco and areca nut use [4]. Human papillomavirus (HPV) is a major driver of the global surge in oropharyngeal cancer [5].

Diagnostic or therapeutic delays in HNC have measurable adverse effects on patient outcomes [6]. Cancer registry data from all over the world show that the majority of HNC cases are diagnosed at an advanced stage [2]. More than half of the HNCs are diagnosed in UICC stage III or IV. Stage IV is the most common stage at diagnosis for oral and oropharyngeal cancer, whereas stage I is the most common for laryngeal cancer [2]. Advanced disease pertains to poorer outcomes in terms of mortality and morbidity [7]. For instance, in oral cavity cancer, early-stage tumors give an 80% 3-year survival rate, whereas late-stage disease shows only 50% survival at 3 years [8]. Hence, screening and early detection of HNC will save lives, reducing in parallel the morbidity and cost intrinsically associated with management of advanced lesions.

The challenge is that patients with HNC present with a large variety of non-specific symptoms that mimic benign conditions as well, such as sore tongue, pain in the throat, odynophagia, dysphonia, unilateral blocked nose, or lump in the neck [6]. The European Head and Neck Society started the Make Sense Campaign (MSC) in 2013, which focuses on encouraging general practitioners to refer a patient to an HNC specialist if the patient complains of one of these six symptoms for more than 3 weeks [9]. The effectiveness is unclear, and clearly, better screening methods are urgently needed. Another focus group is dentists, as they are in a unique position to be able to detect oral cancer potentially at an early stage during oral/dental examination [7]. Continuing educational programs for dentists in this field are definitely mandatory, but comprehensive programs and studies on their effectiveness are still lacking [10]. Beyond effectiveness, the major question is whether current screening methods, i.e., visual inspection with white light and tactile examination of the oral cavity or head and neck lymph nodes, can be improved with modern imaging techniques of optical biopsy or other better screening strategies. This review will present a comprehensive overview of current screening strategies and their limitations, with a focus on populations and patients at risk of developing HNC. Upcoming new technologies will be presented with a critical consideration of their pros and cons.

## 2. Search Strategy

**Figure 1:**
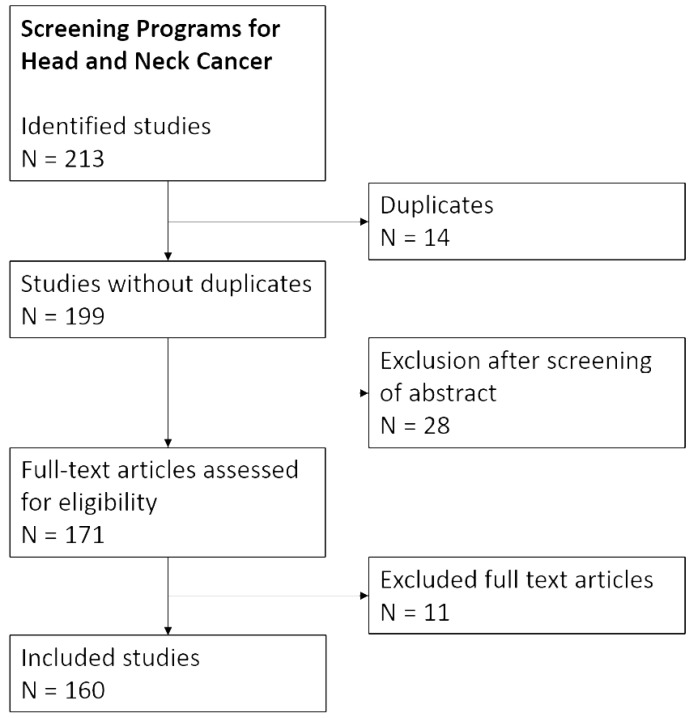
Preferred reporting items for Systematic reviews and meta-analyses (PRISMA) flow diagram of the literature selection process.

As a starting point, a careful online search was performed for clinical guidelines on HNC screening. We could not detect any guidelines published by a national or international scientific society. This was followed by a review conducted in three steps in accordance with the Preferred Reporting Items for Systematic Reviews and Meta-Analyses (PRISMA) guideline [11]. We conducted a literature search for publications in the English language using PubMed and ScienceDirect database (last search: 30 June 2025). The following keywords were used: “screening”, “early diagnosis”, “head and neck cancer”, “oral cancer”, “mouth cancer”, “laryngeal cancer”, “oropharyngeal cancer”, “hypopharyngeal cancer”, “nasopharyngeal cancer”. Hence, this review was focused on the most frequent HNC subsites. We did not look specifically for publications dealing with the screening of cancer of the nose, paranasal sinus, salivary glands, or trachea. Our research retrieved 213 records. Finally, a total of 160 manuscripts were included in the present review based on relevance and scientific evidence. A PRISMA flow diagram of the research is reported in Figure 1.

## 3. Populations and Patients at High Risk of Developing HNC

The primary risk factors associated with HNC include tobacco use, alcohol consumption, HPV infection (for oropharyngeal cancer), and Epstein-Barr virus (EBV) infection (for nasopharyngeal cancer, especially in Asia) [2,12,13,14]. Immunodeficiency, mainly secondary to immunosuppressive treatment after solid organ transplantation and, less stringently, to human immunodeficiency virus (HIV) infection, has also been associated with an increased risk of HNC [15,16,17,18]. Whereas genetic susceptibility to develop HNC in the absence of these risk factors is, in general, very low [19], patients with Fanconi anemia have a 500-fold increased risk of developing HNC, especially oral cancer [20]. Fanconi anemia is an inherited disorder associated with profound DNA repair defects, marked by failure to thrive, congenital malformations, progressive bone marrow failure, and an increased susceptibility to cancer [20]. The inherent DNA repair defect also precludes standard cisplatin-based chemoradiation, markedly constraining therapeutic options for Fanconi anemia patients. HNC tends to appear in these patients at a median age of 30 years, often at advanced stages, with only a 2-year survival rate of 57% [20]. Finally, patients with oral potentially malignant disorders (OPMDs) have an increased risk of developing oral cancer. OPMD includes oral leukoplakia, erythroplakia, oral submucous fibrosis, actinic fibrosis, lichen planus, and discoid lupus erythematosus [21]. Oral leukoplakia, one of the most common OPMDs, has a malignant transformation rate of approximately 2–9.5% [22].

Finally, HNC survivors, especially those who continue to smoke, face a significantly increased risk for developing a metachronous primary tumor, with this risk rising exponentially over time [23,24]. The most important populations at risk are summarized in Table 1.

**Bottom line: high-risk populations**

There are well-defined and less well-defined populations at risk of developing HNC. The associated prevalence of risk factors in patients with HNC varies widely (for instance, adult smoking prevalence in 2020 was 32.6% [25], while Fanconi anemia was set at 0.7%). Seen worldwide, there is a large regional variability of these proportions within the population at risk of developing HNC. This has a significant impact on the optimal and regionally adapted screening strategy (general, invitational, or opportunistic, see below) and its cost-effectiveness.

**Table 1:** Population and patients at risk of developing head and neck cancer (HNC).

Population at Risk	Comment
Smoking	Risk is approximately 10 times higher than that of never-smokers, and 70–80% of new HNC diagnoses are associated with tobacco and alcohol use [26].
Alcohol	Drinking > 3 alcoholic beverages per day was associated with a 1.5–1.7 fold increased risk in men and women [27]. Alcohol and tobacco have a synergistic effect, with the heavy consumption of both increasing HNC risk 40-fold [28].
Human papillomavirus (HPV) infection	Important for oropharyngeal cancer (OPC). The global incidence of HPV positive OPC is increasing, with figures estimating that 25% of OPC cases worldwide are attributable to HPV infection, in contrast to North America, which has a higher prevalence of approximately 65% [29].
Epstein-Barr virus (EBV) infection	Important for nasopharyngeal cancer (NPC). Over 90% of NPC cases, particularly those with undifferentiated types, are EBV-positive [30].
Fanconi anemia	Risk is approximately 500–800 times higher than in the normal population [31].
Immunodeficiency (due to solid organ transplantation or human immunodeficiency virus [HIV])	Incidence rates are 1.5 to 4 times higher in HIV patients than in the general population [32]; it is about 1.4–1.9 times higher in solid organ transplanted patients [33].
Oral potentially malignant disorder (OPMD)	Overall risk for malignant transformation in OPMDs can range from 1% to 34% [34].
Former HNC	Risk of developing a second primary cancer falls within the range of 2–4% per year [23].
Esophageal cancer	5-year cumulative risk of developing second primary HNC ranges from 2.5% to 8.7% [35].
Lung cancer	No numbers published! Vice versa, the risk of developing second primary lung cancer after HNC is about 6% within five years [36].
Occupational exposure	Not part of this review.
Prior irradiation in the head and neck region	Not part of this review.

## 4. Screening for Synchronous or Metachronous Second HNC

Due to the field cancerization theory [37], patients with HNC can have premalignant epithelial changes in other sites of the head and neck, as well as in the upper aerodigestive tract, because of chronic local exposure to tobacco and/or alcohol. This can contribute to the development of synchronous (within 6 months after diagnosis of the index HNC) or metachronous (diagnosed later than 6 months) second tumors [24]. Given the molecular differences in HPV-mediated carcinogenesis, it is reasonable to hypothesize that traditional concepts of field cancerization may not apply to patients with non-smoking, non-drinking HPV-related HNC (almost exclusively oropharyngeal cancer) [38,39]. The rate of second HNC seems, in fact, to be lower in patients with HPV-related HNC [38]. Hence, it may be reasonable to discuss a separate screening program and requirements for patients with HPV-positive versus HPV-negative HNC. This applies equally to HPV-related OPMDs. Follow-up programs for HPV-positive OPMDs would need a different strategy than for HPV-negative OPMDs. However, proposals for such a differentiated screening approach are missing so far.

The traditional standard approach to screening for synchronous second HNC is panendoscopy. Mostly performed under general anesthesia, panendoscopy, also known as triple endoscopy, refers to the use of direct laryngoscopy, esophagoscopy, and bronchoscopy for the workup of patients with HNC [40]. The incidence across studies reporting second synchronous cancer of the aerodigestive tract, i.e., second HNC and other second aerodigestive tract cancer (this is often not differentiated), ranges from 0 to 18% [40]. The pooled incidence of a second HNC in a recent meta-analysis based on 51 studies with 19,914 patients was 4.4% [40]. When patients had a pre-operative computed tomography (CT) scan of the neck or a positron-emission tomography-computed tomography (PET) scan, the panendoscopy did not add value to the therapeutic strategy [40].

HNC patients also have a higher risk for metachronous second HNC at another head and neck subsite separate from the initial primary cancer. In a recent large observational cohort study from the USA, 9% of 1512 HNC patients treated with definitive radiotherapy developed a second primary [23]. The risk increased exponentially with time, with 5-, 10-, and 15-year rates of 4, 10, and 25%, respectively. A quarter of secondary primary cancers were within the head and neck region.

## 5. Screening for the Primary Tumor Site in Patients with HNC of Unknown Primary

A recent systematic review included 41 studies with over 3400 patients to analyze the value of random oropharyngeal biopsies in patients presenting with head and neck squamous cell carcinoma of unknown primary [41]. The primary site identification rate following random biopsies or deep tissue biopsies was less than 5% in most studies. The mean detection rate following ipsilateral tonsillectomy was 34%; two pooled analyses indicated that the mean detection rate following tongue base mucosectomy was 64%, with this figure rising when the tonsillar biopsies were negative. The authors concluded that there is little evidence supporting the practice of random/non-directed oropharyngeal biopsies. Available evidence currently supports palatine tonsillectomy and tongue base mucosectomy compared to deep tissue biopsies [41].

A meta-analysis of 13 retrospective studies, comprising 2623 patients with tonsil cancer or HNC of unknown primary, revealed an overall pooled synchronous contralateral tonsil carcinoma prevalence of 4%, rising to 10% in unknown primary cases [42]. HPV/p16 positivity was associated with synchronous contralateral tonsil carcinoma prevalence of 3%, while HPV/p16 negativity was 8%. The significant prevalence of synchronous contralateral tonsil carcinoma emphasizes the importance of informed discussions regarding contralateral tonsillectomy as a screening method.

**Bottom line for the role of panendoscopy to detect secondary HNC or the primary site in patients with carcinoma of unknown primary**

Panendoscopy is a standard method to detect a synchronous secondary HNC or the primary site in patients with carcinoma of unknown primary. Yield is minimal when comprehensive imaging (neck CT/MRI or PET/CT) has already been completed. Panendoscopy is often needed to confirm the disease based on suspicious findings on initial imaging workup. There has been a lack of diversified strategies for HPV-negative versus HPV-positive tumors, as the risk and the sites of occurrence of second tumors differ between these entities. Concerning metachronous second HNC, risk groups (current smokers and HNC survivors) would probably benefit from screening, but there are no consensus guidelines.

## 6. Screening for Second Primary Cancer at Other Sites of the Aerodigestive Tract in Patients with Index HNC

In a systematic review of 51 studies with 19,914 patients, the overall incidence of a second synchronous primary cancer of the aerodigestive tract was 6.4% [40]. A total of 0.9% and 1.2% of second cancers were found in the esophagus and bronchi, respectively. Among patients who had a prior CT of the neck and chest, a change in management resulting from the second cancer detected through panendoscopy occurred in only 1.1% of cases, and in 0% of cases for those who had a PET scan. Comparatively, the overall rate of major complications from panendoscopy was 0.7%. The authors concluded that the role of panendoscopy in the initial workup of HNC should be limited to confirming suspicious findings from initial CT or PET [40].

The question is whether there are high-risk groups among HNC patients who might benefit from active screening for synchronous or metachronous second primary cancer. Patients with HNC are at a greater risk of developing pulmonary metastases and/or second primary lung cancer [43]. However, it remains uncertain whether lung screening in these patients, when the initial staging studies are negative, confers any survival benefit.

A recent randomized parallel trial of 137 HNC patients indicated that low-dose CT exhibits statistically significant superior sensitivity compared with chest radiography for diagnosing lung metastases and second primary lung cancer. However, there were no significant differences in survival rates shown in this study [44]. A sub-analysis of the National Lung Screening Trial that enrolled patients aged 55 to 74 years with at least a 30 pack-year history of cigarette smoking for chest radiography versus low-dose CT specifically assessed the incidence in HNC survivors [45]. The incidence of lung cancer was higher among HNC survivors compared with participants without HNC (2080 per 100,000 person-years [2.1%] vs. 609 per 100,000 person-years [0.6%]). In HNC survivors, the incidence of second primary lung cancer was 2610 cases per 100,000 person-years in the low-dose CT group versus 1594 cases per 100,000 person-years in the chest radiography group (rate ratio, 1.55), but overall survival was not significantly different [45,46].

After analysis of large clinical trials in the field, the U.S. Preventive Services Task Force (USPSTF) recommended annual lung cancer screening with low-dose CT in a well-defined group of high-risk smokers [47]. A significant proportion of patients with laryngeal cancer meet the introduced criteria. Nevertheless, it is unclear if this implementation of new lung cancer screening guidelines will change clinical practice in the management of patients with laryngeal cancer, and many patients do not receive recommended screening [48]. Further investigation concerning potential barriers to effective evidence-based screening and improved care coordination is warranted [48].

Beyond the lung, HNC patients are at high risk of developing cancer of the esophagus. The prognosis of HNC patients with synchronous esophageal cancer is poor, and therefore early diagnosis of esophageal cancer is of paramount importance for risk stratification and to guide the treatment strategy [49]. A recent review reported a pooled risk of 8.1% of synchronous esophageal cancer [49]. This is a much higher rate than reported in the already mentioned meta-analysis with 0.9% [40]. There may be geographical differences with higher rates in Asia, as well as the fact that tools with different sensitivities have been applied in the two studies. The incidence of a synchronous but mainly metachronous esophageal second primary cancer in 365 HNC patients in a recent series from Taiwan found by panendoscopy was 10.1% [50]. Patients with hypopharyngeal cancer, high alcohol and cigarette consumption had the highest risk. In contrast, this risk seems to be lowest for HPV-positive HNC [51]. It is important to emphasize that early esophageal cancer may be easily overlooked. This might explain the large variety of these prevalence numbers of finding a second primary esophageal cancer in patients with an index HNC.

A recent small study from Portugal showed that an annual screening esophagogastroduodenoscopy in HNC survivors was cost-effective, as esophageal cancer was detected in 10.9% of the patients, and at an early stage [52]. It was proposed that the use of narrow-band imaging (NBI; see also below, innovative imaging technologies) when performing screening esophagoscopy in patients with HNC might improve the detection of early-stage esophageal malignancies [53].

**Bottom line for screening for second primary cancer at other sites of the aerodigestive tract in patients with an index HNC**

With modern imaging, especially when CT/MRI neck and lung imaging or PET-CT are performed as part of the initial tumor staging, the rate of synchronous second tumors, particularly for lung and esophagus, is low. These sites are at risk of developing metachronous tumors during the follow-up. Certain at-risk groups may benefit from active screening; for instance, patients with hypopharyngeal cancer should be screened for esophageal cancer, or HNC survivors who are heavy smokers should be recommended for screening for lung cancer. There is a lack of prospective studies evaluating the optimal method and frequency for such screening in high-risk groups.

## 7. Screening for Second HNC in Patients with Other Index Aerodigestive Tract Cancer

Similarly, patients with esophageal squamous cell carcinoma frequently develop second primary tumors in the upper aerodigestive tract, most often in the head and neck region [54]. The 5-year cumulative occurrence rate of second HNC is around 3–10% [55,56]. Based on studies mainly from Asia, most of the second HNC in patients with an index esophageal cancer are located in the hypopharynx [49,57]. A study of 439 superficial esophageal cancer patients reported that 53 metachronous HNC cancers developed in 9.1% of patients after a median follow-up period of 46 months, and the cumulative incidence rates of metachronous HNC at 3, 5, and 7 years were 5.3%, 9.7%, and 17.2%, respectively [56]. A systematic review of 6483 esophageal patients from 12 studies in Japan revealed a pooled prevalence of secondary HNC of 6.7%, including 48.2% synchronous and 51.8% metachronous HNC, 85.3% at an early stage, and 60.3% located in the hypopharynx [57].

Lung cancer screening is discussed for patients with HNC, but patients with lung cancer may not be screened for HNC despite the common risk factor of smoking for both cancers. Interestingly, we could not identify any study looking for a second HNC in patients with an index lung cancer.

**Bottom line for screening for second HNC in patients with index esophageal or lung cancer**

Future research should evaluate whether reciprocal screening strategies (e.g., HNC surveillance in esophageal or lung cancer patients) could improve outcomes and cost-effectiveness.

## 8. Screening for Early Detection of Recurrent HNC

Detection and management of local and distant HNC recurrences remain a major challenge. A well-structured follow-up strategy including a clinical examination, endoscopy, and imaging is essential, with a focus on detecting recurrences and managing late treatment effects within the first 2 years after treatment [58]. Ultrasound seems to have a high positive predictive value (PPV) of about 96% and a negative predictive value (NPV) of 93% to detect a neck recurrence when performed by a skilled practitioner. The PPV/NPV for CT, MRI, diffusion-weighted MRI, and PET-CT are about 62%/92%, 25%/94%, 89%/100% for T classification (70%/96% for N classification), and 42%/98%, respectively. Hence, ultrasound is reliable in experienced hands. CT has a low PPV, diffusion-weighted MRI has a better PPV than standard MRI, and PET/CT has an excellent NPV, but low PPV [59,60,61]. Another relevant question is whether routine surveillance of the patients is associated with a survival benefit compared with those who presented with symptoms [60]. Hence, a more differentiated follow-up strategy might also take into account the prognosis of the patient, comorbidities, and risk factors [60]. The landmark study of Mehanna et al. analyzed the role of PET-CT image-guided surveillance as compared with planned neck dissection in the treatment of patients with HNC who have advanced nodal disease (stage N2 or N3) treated with chemoradiotherapy [62]. The question was whether PET-CT follow-up could help to avoid neck dissections. PET-CT-guided surveillance resulted in fewer neck dissections (54 vs. 221 planned neck dissections) with similar survival rates between the two groups. Hence, PET-CT-guided surveillance resulted in considerably fewer operations and was more cost-effective in patients with advanced nodal disease undergoing radiochemotherapy [62].

Forty-eight studies on post-treatment surveillance imaging in HNC were evaluated in a recent systematic review [63]. The analysis revealed that almost half of the cases of locoregional recurrences and/or metastases (40.9%) were only detected by imaging. The mean time of detection of recurrent or metastatic disease was 11.5 months. Most included studies reported superior results with PET-CT when compared to other imaging techniques [63]. The authors concluded that a systematic imaging surveillance in locoregional advanced HNC during at least one (preferably 2) year(s) after treatment should be recommended. Most recently, long-axial field of view (LAFOV) PET-CT systems have been introduced onto the market. LAFOV PET-CT scanners can capture a larger volume of data simultaneously, resulting in higher sensitivity [64]. A first retrospective study analyzed LAFOV PET-CT results in HNC patients [65]. There was some improvement for tumor lesion detection, but it is too early to say if this will translate to improved clinical performance in PET-CT post-treatment surveillance.

Most guidelines recommend 1–3 monthly follow-ups in the first year after treatment, 2–3 monthly visits for the second year, and 4–6 monthly visits in years 3–5 [60], but the impact of the frequency of follow-up visits has not been studied in prospective trials. A population-based study using electronic health care data relating to 5310 treated HNC patients from Ontario during 2007–2012 revealed no impact of the frequency of visits on the survival of the patients [66]. A recent critical appraisal of current guidelines concluded that there is no rationale for applying the same follow-up program to each of the HNC subsites, because the subsites have different risks and timing of recurrence and second primary tumors [67].

**Bottom line for screening for early detection of recurrent HNC**

To date, most guidelines recommend fixed intervals for follow-up, at least for the first 3–5 years, independent of the primary HNC subsite. Future research should focus on risk stratification, the value of symptom-free detection of recurrences, and the active role that patients might play in determining their own follow-up regimen [67].

## 9. Evaluated Primary HNC Screening Programs

Different types of screening programs must be distinguished from each other. An invitational screening program might involve sending out letters to a defined population group, inviting them to participate in a health check. An opportunistic approach might involve screening individuals who are already attending a doctor for another reason. The most important screening strategies are summarized in Table 2.

The National Health Service (NHS) England reviewed in 2019 the current screening programs in the country [68]. Among HNC, oral cancer is mentioned in one sentence only. Although dentists could screen for oral cancer during routine check-ups, oral cancer screening is not part of the national screening programs.

In France, especially in the north-west region, where they have one of the highest incidence rates of HNC in Europe [69,70], patients with the diagnosis of HNC in 2010 were analyzed within four French cancer registries with regard to medical consultations during the year prior to diagnosis and their impact on the stage of cancer at diagnosis [71]. Frequent general practitioner visits correlated significantly with earlier, localized HNC diagnoses. The visit rates to dentists were very low. The authors concluded that high-quality professional training for general practitioners is necessary to improve early detection of HNC. Nevertheless, the benefit of such early detection remains to be shown, and the target population in high-risk regions must be defined [71].

The USPSTF updated their recommendation in 2013, reviewing the evidence on whether screening for oral cancer reduces morbidity or mortality and on the accuracy of the oral screening examination for identifying oral cancer or OPMDs that have a high likelihood of progression to oral cancer in asymptomatic adults aged ≥ 18 years (neither in high-risk adults, i.e., those over the age of 50 who use tobacco or for average risk adults in the general population) who are seen by primary care providers. This recommendation does not focus on screening of the oral cavity performed by dental providers or otolaryngologists. The USPSTF concluded that the current evidence is insufficient to assess the balance of benefits and harms of screening for oral cancer in asymptomatic adults [72].

Another relevant point is that patients at high risk for oral cancer demonstrate limited awareness of such a disease, knowledge of causative risk factors, and other conditions associated with alcohol and tobacco use [73]. Efforts to screen this high-risk demographic in the United States have proven unsuccessful due to poor attendance of high-risk males in free oral and HNC screening programs [74,75]. A shift away from office-based screening to a community-based approach in which trained health workers actively seek out high-risk males to undergo screening may be more successful [74,76]. Hospital-based screening campaigns seem to attract individuals with prior HNC or a history of cancer outside the head and neck region, whereas community-based screening events tend to attract participants with general risk factors such as smoking or alcohol abuse [77].

One should be aware that not only men fall under the risk group of people who smoke, drink alcohol, or both, but also women, depending on the cultural and social habits. Five hundred and ten (510) cancer-free women admitted to the internal medicine service at a U.S. academic center were enrolled to participate in a monocenter study from 2014 to 2017 [78]. Among these, 370 (73%) women were at high risk for developing oral cancer, defined by smoking status, alcohol use, or both; 57% of these high-risk women reported having no primary dentist. All high-risk women received bedside smoking cessation counseling, oral cancer informational handouts, and were offered oral screening examinations during hospitalization. Only 41% of these women took advantage of these oral cancer screening examinations during the hospital stay, but 66% of high-risk patients discussed oral cancer screening with their primary care physician after the hospitalization. Hence, HNC awareness can be improved in high-risk women, and screening by a physician when admitted for other reasons, but fulfilling high-risk criteria for developing HNC might be an alternative screening option to be studied.

However, one should keep in mind that the prevalence of HNC is unevenly distributed across world regions, and Southeast or South Asian and Melanesian males comprise the highest-risk population [79]. The survival rates are also very different [80]. For instance, the 5-year survival rate in North America is 75–93% for localized (stage I/II) tumors, with decreases to 20–52% when regional or distant metastasis occurs [81,82]. In low-resource countries, such as India, the survival difference is even more extreme, with a survival rate of about 60% in the early stage versus 3% in advanced disease [83]. Of course, the reasons seem to be multifactorial. Beyond disease stage, differences in access to health care, different healthcare systems, and treatment options may influence the survival rates.

Hence, in regions of the world with a high incidence of HNC, general screening strategies may be effective. For instance, a large cluster-randomized controlled screening trial with 96,517 eligible participants in Kerala, India, i.e., an area with very high incidence of oral cancer, reported in 2005 a significant reduction in mortality for screened male tobacco and/or alcohol users undergoing three rounds of screening by oral visual inspection by trained health workers at 3-year intervals from 1996 to 2004. The mortality rate of unscreened high-risk males was 42.9 per 100,000 compared to 24.6 per 100,000 in the screened arm, yielding a mortality rate ratio of 0.57 (95% confidence interval [CI] 0.35–0.93 [84]. These results cannot be extrapolated to other subsites, as only the oral cavity can be examined by trained health workers or by a general practitioner without special equipment. One can conclude that screening of asymptomatic persons with high-risk characteristics in primary care settings may only be effective if the prevalence of oral cancer as an index tumor is high. To our knowledge, this is the only level 1 evidence study regarding the efficacy of screening for HNC [85]. Although this trial provides level 1 evidence, the generalizability to non-Asian populations with a lower prevalence of oral cancer remains uncertain. The involved health workers received special training to detect oral cancer via oral inspection. The importance of such specialized training—and in other high-risk regions, possibly also of clinicians for better screening—should be investigated in future studies.

In 2023, the International Agency for Research on Cancer (IARC), i.e., the specialized cancer agency of the World Health Organization WHO, published a 370-page handbook on oral cancer prevention [86,87]. The panel, comprising specialists from around the world, thoroughly reviewed the current evidence to provide recommendations that could be applied globally. Key measures for significantly reducing the risk of oral cancer include avoiding or stopping tobacco smoking and the usage of areca nuts, including betel quid with or without tobacco, and reducing alcohol consumption. The handbook brings together current evidence on both primary and secondary prevention to detect precancerous lesions or early-stage cancer.

Furthermore, screening methods, training needed, performance guidelines, and data on sensitivity and specificity are summarized [88]. The WHO commented that screening does not need to be performed by a physician. Successful programs would have been run by other trained healthcare workers. Trained nurses would also effectively recognize red-flag symptoms in a patient’s history and signs in an oral examination. Irrespective of the professional chosen to do the screening, it would be essential that they are experts with knowledge of how to perform an oral examination, assess risk factors, educate the patient, and promote healthy habits. There must also be a clear and reliable pathway to follow if a suspicious head and neck lesion is found [88]. Nevertheless, the IARC can only present a few studies in the summary that prove the effectiveness of screening measures.

Concerning special risk groups, a retrospective analysis of a screening of all liver transplant candidates with a recent history of smoking combined with daily use of alcohol prior to a 6-month sobriety period from 1999 to 2009 was evaluated [15]. Among 581 patient evaluations performed by the otolaryngologist for HNC screening prior to liver transplantation, one (0.17% of evaluations) case of HNC was detected over the 10-year period. Given the consumption of resources required for this screening strategy and the limited yield, the authors concluded that current screening internal guidelines are ineffective and need to be reconsidered.

**Bottom line for the results of the evaluated large HNC screening programs**

There have been many efforts in the past to establish HNC and oral cancer screening programs in particular, but so far, the effectiveness has not been proven as yet, even for high-risk groups.

## 10. Efficacy and Cost-Effectiveness Modeling of HNC Screening

Early diagnosis by screening asymptomatic individuals can reduce the cost of treatment [89]. Studies reporting on the cost-effectiveness of cancer screening usually account for quality-of-life losses and healthcare costs owing to cancer [90].

In 2006, the U.K. Health Technology Assessment (HTA) program analyzed eight strategies on the cost-effectiveness of screening for oral cancer in primary care [91]: (A) no screen; (B) invitational screen—general medical practice; (C) invitational screen—done by general dental practice; (D) opportunistic screen—by general medical practice; (E) opportunistic screen—by general dental practice; (F) opportunistic high-risk screen—by general medical practice; (G) opportunistic high-risk screen—by general dental practice; and (H) invitational screen—by specialist. The main measures were mean lifetime costs and quality-adjusted life-years (QALYs). Strategies B, C, E, and H were dominated (or extended dominated) and therefore not cost-effective. Overall, only high-risk opportunistic screening by a general dental or medical practitioner (strategies F and G) may be cost-effective. So far, however, the NIH has not incorporated these results into any routine oral cancer risk assessment by dentists or general practitioners.

As nasopharyngeal cancer is endemic in a few well-defined populations and as early-stage nasopharyngeal cancer is highly curable, the design of an optimal screening program for nasopharyngeal cancer has been discussed for a long time. Anti-EBV IgA antibody (e.g.,—early antigen [EA-IgA], anti–EBV capsid antigen [VCA-IgA], and anti–EBV nuclear antigen 1 [EBNA1-IgA]) serological testing is commonly used to detect asymptomatic nasopharyngeal carcinoma [92]. Furthermore, individuals with detectable plasma EBV DNA but without an immediately identifiable nasopharyngeal cancer seem to be more likely to have the cancer identified in another round of screening performed 3 to 5 years later [93]. Nevertheless, the test’s low sensitivities and specificities impede its use in nasopharyngeal carcinoma screening in asymptomatic participants [13,94]. Due to the latest Cochrane review on this topic published in 2015, no data from randomized controlled trials (RCTs) or clinical controlled trials (CCTs) were available to allow for determining the efficacy of screening for nasopharyngeal cancer, or the cost-effectiveness and cost-benefit of a screening strategy [95]. A systematic review of 13 studies revealed that general testing in Asian Americans aged 50 years or in general for men aged 50 years for circulating tumor EBV DNA (ctDNA EBV-DNA) for nasopharyngeal cancer is not cost-effective [96]. However, the effectiveness could be increased in some specific circumstances. A recent review focused on family members of nasopharyngeal carcinoma patients showed that familial screening using EBV serology may facilitate early nasopharyngeal cancer detection in endemic areas [97].

Dedhia et al. transposed the data from the Kerala study into a Markov model to calculate costs of a community outreach oral cancer screening program using oral examination and biopsies if needed for males more than 40 years regularly using tobacco and/or alcohol in the United States [76]. They calculated the difference in costs and QALYs between the non-screened and screened people. The not-screened arm was dominated with an incremental cost of USD 258 and an incremental effectiveness of −0.0414 QALYs. Using the 75,000 USD/QALY metric, the maximum allowable budget for a screening program equaled USD 3363 per screened person over a 40-year time course. It was concluded that a community-based screening program targeting high-risk males is likely to be cost-effective [76].

In contrast, such a program can be cost-effective in India. Dwivedi et al. used a Markov modelling to estimate the cost and health outcomes of four different approaches (conventional oral examination (COE), toluidine blue staining (TBS), oral cytology (OC), and light-based detection (LBD) for screening oral cancer over time from a societal perspective and QALYs in two scenarios: mass-screening strategy, or a high risk screening strategy versus no screening [98]. Mass-screening using LBD at three years had the fewest incident cases (3271.68 new cases per 100,000 population) and averted the maximum number of oral cancer deaths (459.76). High-risk screening using COE at 10 10-year interval incurred the least lifetime cost of USD 2,292,816.21 (INR 182,794,468.26). The high-risk strategies (USD/QALY), namely COE 5 years (−29.21), COE 10 years (−90.68), TBS 10 years (−60.54), and LBD 10 years (−13.51), were dominant over no screening. Hence, the most cost-saving approach was the COE at an interval of 10 years for oral screening in high-risk populations above 30 years of age [98]. In India, tertiary care institutions face the challenge of delivering specialized services while simultaneously grappling with primary care responsibilities, inadequate manpower, and laboratory facilities [99,100]. Mobile health (mHealth) technologies applied by health workers, which allow remote expert consultations, might help bridge this gap by facilitating the diagnosis of screen-positive HNC individuals who cannot access in-person specialists [101]. A prospective study enrolled 10,101 high-risk individuals from rural settings of Varanasi district, India, between 2021 and 2023 for such an mHealth approach, taking smartphone white light images of the oral mucosa. Prevalence of substance use was 55.7%, with 21.4% having OPMD or oral cancer. Sensitivity of field workers and remote diagnosis for detecting OPMD or oral cancer was moderate (about 66%) when compared with an onsite expert, but the high specificity (96%) may make mHealth a valuable tool for improving oral cancer screening coverage in rural areas if cost-effectiveness could also be shown [101].

Markov modelling was also used to simulate costs and QALYs of both the screening and no-screening programs in the Thai population (in which the incidence of HNC is lower than in Europe, with 8.8 per 100,000), aged over 40 years [102]. The types of examination considered were (1) mouth self-examination (MSE), (2) visual examination by trained dental nurses (VETDN), (3) visual examination by trained dentists (VETD), and (4) visual examination by oral surgeons (VEOS). The screening program yielded higher costs (USD 41) and QALYs (0.0044 years) than the no-screening program, producing an incremental cost-effectiveness ratio (ICER) of about USD 9340 per QALY gained. This indicated that a screening program is cost-ineffective in the Thai context, where the cost-effectiveness threshold is about USD 4800 per QALY gained.

**Bottom line for the cost-effectiveness of HNC screening programs**

Most analyses of general screening programs have been unable to demonstrate a favorable cost-effectiveness ratio; however, programs for selected at-risk groups could be cost-effective.

## 11. Awareness Campaigns

**Figure 2:**
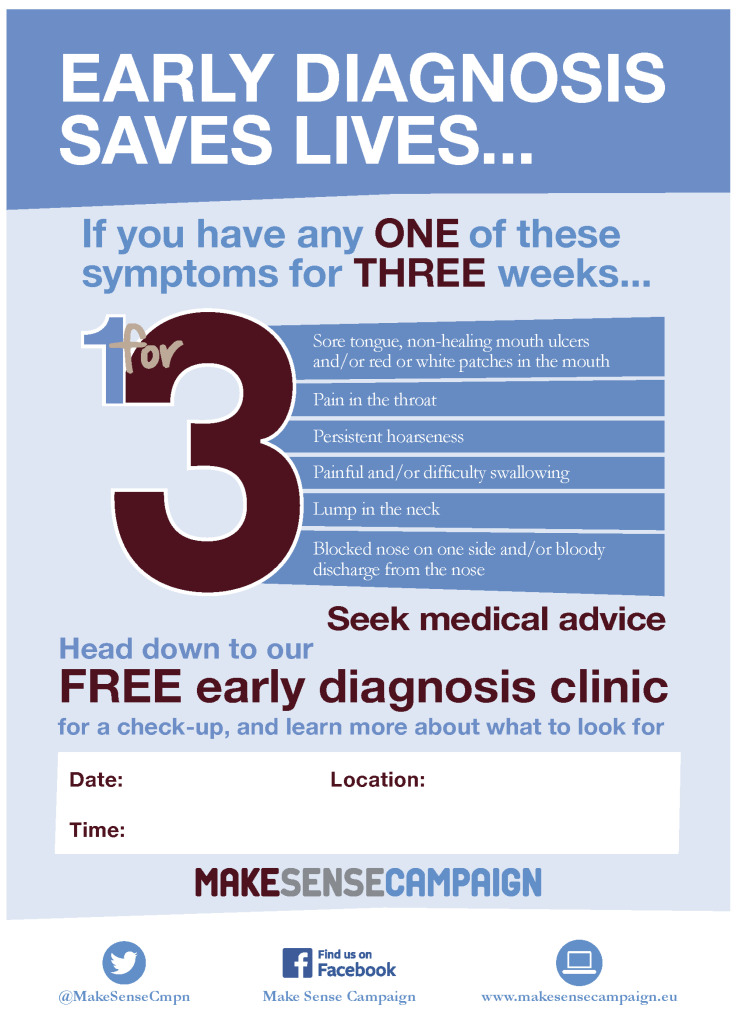
Make Sense Campaign Poster. The Make Sense Campaign of the European Head and Neck Society offers freely available campaign resources (https://makesensecampaign.eu/en/about-the-campaign/resources/ (accessed on 17 August 2025)) like this poster.

Although HNC is the sixth most common type of cancer in Europe, there is low awareness of HNC among the general public and the healthcare community in Europe. In light of this and the ongoing focus on cancer as a top health priority for the European Union, the multi-channel, global awareness campaign called “Make Sense campaign” (MSC) [9], in partnership with the European Head and Neck Society (EHNS), the European Cancer Patient Coalition (ECPC), and the Parliamentary Intergroup on Cancer, was started in 2013 (Figure 2). The goal is to raise awareness of HNC and ultimately improve outcomes for patients. MSC published a white paper (last update in 2020) and set out a plan of action to drive change for HNC patients in Europe [103]. Important aims are the active engagement of participating hospitals in prevention strategies for HNC and to support early referrals to qualified healthcare professionals. The European population is addressed via social media. Participating hospitals organize an annual early diagnosis screening as a free drop-in screening session open to the local population. This is typically advertised in the department, but it is also used on social media to publicize it to a wider audience [85]. Between 2013 and 2021, about 112,000 people were screened during an MSC [104].

An oral cancer awareness campaign in Northern Germany was carried out from 2012 to 2014, including pre-, post-, and process evaluations [105]. The target group was elderly, educationally disadvantaged male citizens ≥ 50 years. The awareness within the target group was significantly increased. Media coverage showed that regional media adopted the topic of oral cancer and placed it on their published agenda. If this is all followed by more effective and earlier detection of oral cancer, it remains an open question [106].

**Bottom line for HNC awareness campaigns**

Awareness campaigns on HNC should be promoted and supported by head and neck surgeons, radiotherapists, oncologists, and other physician groups involved in the treatment of HNC patients. The effects of detecting HNC earlier are unknown.

## 12. Liquid Biopsy Strategies

Liquid biopsy means the analysis of tumor material (for example, cells, nucleic acids, or other molecules) obtained in a minimally invasive or non-invasive manner through the sampling of blood or other body fluids. The concept should especially work well for virus-associated tumors [107,108].

Research in liquid biopsy is the most advanced for EBV-positive nasopharyngeal cancer, especially in Southern China and Southeast Asia [13]. Several EBV-encoded oncoproteins, which are therefore optimal targets for a liquid biopsy analysis, control the carcinogenic properties of the virus, from oncogenesis to progression to nasopharyngeal cancer [105,106]. Several large studies, mainly from Asia, have shown that detectable EBV ctDNA in plasma during follow-up indicates tumor recurrence, while undetectable EBV ctDNA indicates continuous remission [109,110,111]. Among the patients with detectable EBV ctDNA who develop a recurrence, the positive EBV ctDNA results preceded radiological and/or clinical evidence of disease recurrence by several months. It has been suggested that post-surveillance screening based on plasma EBV ctDNA may be used instead of PET screening for high-risk patients. This might result in cost savings of approximately 80% by performing 2–4 blood tests per year instead of an annual PET [110]. As a consequence, both the National Comprehensive Cancer Network (NCCN) and European Society for Medical Oncology (ESMO) guidelines already recommend evaluating EBV ctDNA at least every year after treatment of nasopharyngeal cancer [112,113]. Moreover, additional circulating free DNA (cfDNA) methylation testing in combination with EBV ctDNA screening might even help to further improve the positive predictive value [114].

The issue of liquid biopsy is becoming increasingly important for HPV-positive tumors due to the high prevalence of these lesions. Liquid biopsy may not only offer a highly reliable and accurate screening tool to early detect tumor recurrence, but may also be an early way to screen for HPV-associated HNC in otherwise healthy people before the development of symptoms [115]. ctHPV16 assessed in liquid biopsy, when using a plasma next-generation sequencing (NGS)-based approach, seems to have a high sensitivity and specificity to discriminate between HPV-positive HNC patients and controls [116,117]. Using blood samples from patients who were healthy at the time of blood draw, but then years later developed HPV-positive HNC, it has been shown that this assay can predict for HPV-positive oropharyngeal cancer in patients with a 79% overall sensitivity and 100 percent specificity within 4 years of diagnosis and a maximum lead time of 7.8 years [118].

The De-escalated Adjuvant Radiation Therapy (DART) phase 3 RCT showed that in patients with HPV-associated oropharyngeal squamous cell carcinoma, postoperative minimal residual disease (MRD), detected through ctHPV DNA, was associated with a higher risk of disease progression [119]. Moreover, a recent secondary analysis of the DART trial found that when added to the pathologic factors considered, ctHPV DNA assessment may improve the selection of patients for treatment de-escalation. In addition, the 3-month post-treatment time point, early in surveillance, may identify a sizable portion of patients with progression and may guide intervention and surveillance after surgery for HPV-associated oropharyngeal squamous cell carcinoma [120]. There are new developments, like NGS-based ctHPV DNA assays that seem to outperform currently available ctHPV DNA assays in MRD detection, identifying about twice as many patients with MRD (80% versus 40% sensitivity) [121,122,123].

The accuracy of ctDNA testing for HPV-positive tumors already seems to be very good (high positive predictive value > 95% as well as high negative predictive value of 100% for identifying disease recurrence in patients with HPV-associated oropharyngeal cancer [124]). If further validation studies with different assays confirm these results, the question arises as to how it can be applied optimally and in the best personalized way in patient aftercare. Initial model calculations have shown that monthly testing could be cost-effective, as it reduces conventional aftercare, and the early detection of a recurrent tumor saves costs [125].

It should also be considered how promising liquid biopsy strategies are for non-virus-associated HNC. Genomic profiling using next-generation sequencing (NGS) panels has identified some mutated genes in both HPV+ and HPV- HNC patients, including PIK3CA, TRAF3, TP53, NOTCH1, and CDKN2A [126,127,128]. However, the number of studies and the follow-up period are still too small to allow for a conclusive evaluation of the methods.

Another interesting strategy could be to combine a liquid biopsy strategy with imaging. The DETECT-A (Detecting cancers Earlier Through Elective mutation-based blood Collection and Testing) study used a multi-analyte test, called CancerSEEK, to screen 10,006 women not previously known to have cancer [129]. CancerSeek uses protein biomarkers and genetic markers to screen for ovarian, liver, stomach, pancreas, esophagus, colorectal, lung, or breast cancer [130]. Positive blood tests in the DETECT-A trial were independently confirmed by a diagnostic PET-CT, which also localized the cancer. Only 1.0% of participants underwent PET-CT imaging based on false positive blood tests, and 0.22% underwent a futile invasive diagnostic procedure [129]. Hence, this combination of liquid biopsy and PET-CT seems to be very promising for early cancer detection [131]. This approach has not yet been applied in prospective clinical trials for HNC screening. Recently, a small prospective trial on 41 HNC patients undergoing radiochemotherapy had serial blood testing using an NGS panel for HPV and somatic mutations in combination with serial PET-CT [132]. The liquid biopsies were better at determining true disease status than PET-CT post-treatment (liquid biopsy: sensitivity and specificity of 83% and 95% respectively, versus 67% and 42% respectively, for PET-CT).

**Bottom line for liquid biopsy strategies for HNC screening**

Liquid biopsy strategies are at the moment the most promising new strategies to improve primary and secondary HNC screening, especially for EBV and HPV-related HNC. More clinical trials showing effects on earlier HNC or recurrence detection are on the way. Still more experimental are trials to analyze whether adjuvant therapy should be adapted based on the detection of MRD.

## 13. Innovative Imaging Technologies

At an HNC consult, white light examination is the standard for the clinical assessment. This is combined with biopsies of HNC suspicious lesions, followed by pathological examination as the gold standard for definitive diagnosis. This is subject to overtreatment for benign lesions. Hence, the clinical examination and its evaluation should be conducted with caution. At best, an experienced clinician performs the examination. Especially, as there is still no innovative method with higher reliability in clinical use, if there were a reliable optical method, a biopsy or even an (unnecessary) resection could be avoided in these cases in the future. Second, at the moment, the examiner only receives information in front of places that were biopsied. Hence, the method contains an important selection bias. An optimal optical method would theoretically yield an infinite number of measurements. In general, diagnostics based on biopsies are time-consuming and invasive, which is not suitable for multiple examinations for patients who need long-term follow-up or those affected by OPMD, requiring lifelong surveillance. Hence, there is an urgent unmet need for real-time, non-invasive diagnostic methods. Older adjuvant screening tools to support the standard otolaryngology examination are presented in Table 3.

At present, various non-invasive optic biopsy methods that can also be used for screening are emerging and gaining attention (Table 4) [144]. The spectrum ranges from spectroscopic techniques, in vivo in situ microendoscopy, narrow-band imaging (NBI), and fluorescence imaging, to optical coherence tomography (OCT) [145].

Most data are published about NBI, using blue and green lights with wavelengths at 415 and 540 nm, able to penetrate the mucosal surface and to be absorbed by the superficial blood vessels highlighted in dark blue or brown. Using standard videoendoscopic screening with white light combined with NBI during routine follow-up of 206 patients treated with radiotherapy in a tertiary HNC center, identified 68 lesions by this combined approach. Of these, 62 were histopathologically confirmed to be neoplastic. The rates of detecting cancerous lesions by white-light and NBI modes were 100% and 97% for oral lesions, 69% and 100% for oropharyngeal lesions, and 39% and 100% for hypopharyngeal lesions, respectively. For overt T1–T4 lesions, NBI and white light showed comparable detection rates. However, NBI mode was significantly better than white-light mode for the detection of carcinoma in situ [165]. Better might be the use of NBI classification systems; NBI allows a more distinct image of the subepithelial capillary system [148]. This led to the intrapapillary capillary loop (IPCL) classification, allowing for the classification of abnormal oral squamous epithelium changes [166]. A recent meta-analysis showed that defining IPCL II and above as a clinically positive result demonstrated the most optimal overall diagnostic accuracy for the malignant transformation of OPMDs, with a sensitivity and specificity of NBI of 0.87 and 0.83 [148]. So far, cost-effectiveness studies are missing.

Several new optical biopsy techniques are in preclinical testing to analyze the mucosa of the upper aerodigestive tract for cancer screening without the need to take a real biopsy. A pilot study used in vivo Raman spectroscopy for the detection of oral neoplasia on 28 healthy volunteers and 171 patients having various lesions of the oral cavity. The final algorithm based on comparisons to histology resulted in a sensitivity and specificity of 94% in discriminating normal from abnormal spectra of precancerous lesions and oral cancer [167]. These results were validated by other researchers [168]. Raman spectroscopy was also applied to screen saliva, so far only in a few patients [169], but it seems that some spectra might discriminate OPMD patients from normal controls. Beyond analysis of biomaterial characteristics, OCT also allows for visualizing small lesions. A preliminary study on eight healthy probands, but also patients with 12 hyperplastic, 11 dysplastic, and 4 early-stage cancerous lesions, revealed a sensitivity, specificity, positive and negative predictive value to detect dysplasia and early-stage cancer of 100%, 95%, 94%, and 100%, respectively [170].

Biophotonic techniques are not only of interest for primary HNC detection. Detecting secondary tumors due to field cancerization is another task. Early detection of field cancerization in an easily accessible location of the upper aerodigestive tract, such as the buccal mucosa, could potentially be used to screen for second HNC or for lung cancer [171,172]. Using probe-based reflectance spectroscopy in vivo to screen for changes in the optical properties of the buccal mucosa of 23 HNC patients and 23 non-oncologic controls identified oncologic patients with a sensitivity of 78% and a specificity of 74% [160].

Finally, the integration of artificial intelligence into image analysis and liquid biopsy data represents an emerging frontier in HNC screening. Machine learning algorithms can enhance diagnostic accuracy, reduce false positives, and facilitate the interpretation of large volumes of clinical and molecular data. The development of digital platforms that combine artificial intelligence with telemedicine tools could expand access to screening programs in rural or resource-limited areas.

Looking forward, the true potential of next-generation screening may not lie in any single technology, but in its synergistic integration. Liquid biopsies and advanced optical imaging should be viewed as complementary rather than competing tools. A future, highly effective screening paradigm could involve a two-step process; an initial, minimally invasive liquid biopsy (e.g., ctDNA testing for HPV or EBV) could be deployed broadly among high-risk populations to detect molecular evidence of cancer. Individuals who test positive would then undergo a targeted examination with advanced, non-invasive imaging techniques like NBI or OCT to precisely localize and characterize the suspicious lesion in vivo. This integrated approach could significantly improve diagnostic yield and cost-effectiveness by reserving more resource-intensive imaging procedures for patients with the highest probability of disease, representing a pivotal shift towards a more precise and personalized screening strategy.

**Bottom line for innovative imaging technologies for HNC screening**

Next to liquid biopsy strategies, innovative biophotonic techniques will drive improvements in HNC screening. When such technologies obtain a consistent accuracy comparable to that of histology, this will allow us to design completely new screening strategies, especially for individuals at high risk of developing HNC.

## 14. Conclusions

Despite the declining global smoking prevalence, HNC incidence continues to rise and remains a major public health challenge. Early detection is critical to improving outcomes, yet most cases are still diagnosed at an advanced stage. Traditional HNC screening methods, such as visual inspection and panendoscopy, have limitations in sensitivity, cost-effectiveness, and applicability. High-risk populations, such as tobacco and alcohol users, and those with genetic disorders like Fanconi anemia, require tailored screening strategies. Current evidence does not support widespread screening in general asymptomatic populations, but targeted approaches, especially for oral cancer, show promise in high-prevalence regions like South Asia. Community-based screening, especially when led by trained health workers, has demonstrated success in certain settings. Liquid biopsy techniques for EBV and HPV-related HNCs show great promise for non-invasive, early detection and recurrence monitoring. Similarly, innovative imaging tools, for instance NBI, Raman spectroscopy, and OCT, offer encouraging diagnostic potential, especially for head and neck regions that are easier to reach, i.e., mainly the oral cavity. However, many of these technologies remain in their experimental stages and require further validation before they can be widely implemented.

However, translating these technological advances from research to routine clinical practice involves overcoming substantial hurdles. For technologies like liquid biopsy and AI-assisted optical imaging to be implemented effectively, several key steps are necessary. These include the establishment of standardized protocols for sample collection and analysis, securing regulatory approval from health authorities, and demonstrating cost-effectiveness to justify their integration into healthcare systems. Moreover, extensive education and training will be required for clinicians to ensure they can proficiently use these new tools and accurately interpret their results, ensuring that their diagnostic potential leads to tangible improvements in patient care.

Despite numerous awareness campaigns, public and healthcare provider knowledge about HNC symptoms and risks remains insufficient. There is an urgent need for effective implementation of educational programs, particularly targeting dentists and primary care providers. Screening for second primary tumors and recurrences remains complex, especially in the absence of standardized risk-stratified follow-up protocols. Cost-effectiveness analyses largely support opportunistic, high-risk patient screening over mass population programs. These findings underscore the imperative for region-specific, evidence-based screening frameworks. Future research should focus on stratified approaches using advanced diagnostics and risk profiling to enhance both efficiency and patient outcomes. In conclusion, an integrated, technology-driven, and risk-based screening model holds the most promise for improving HNC detection and survival globally.

## Figures and Tables

**Table 2:** Current head and neck cancer (HNC) screening strategies.

Strategy Type	Method	Use Case/Notes
Opportunistic	Oral visual/tactile exam	Dentist and physician visits
Invitational	Letter-based outreach	Rarely implemented; mainly by otolaryngology departments used
Community-based	Trained health workers conduct screenings	With health workers mainly studying in India
Hospital-based campaigns	Drop-in screenings	Like in the European Make Sense Campaign
Self-examination	Mouth self-exam	Not yet widely validated

**Table 3:** Advanced but more traditional techniques to screen for head and neck cancer (HNC), mainly used in vivo for oral cancer (overview in [88]).

Technique	Comment
Oral cancer screening with smartphone images from the field to specialists	Sensitivity of 82–94%; specificity of 72–100% [101,133,134,135,136]
Vital staining	With toluidine blue or Lugol’s iodine: pooled sensitivity of 86% and specificity of 68% [137,138]
Cytology	As exfoliative biopsy or brush biopsy cytology; pooled sensitivity of 90–92% and specificity of 94% [139,140]
DNA cytometry	Mainly to detect aneuploidy; pooled sensitivity of 76% and specificity of 98% [141,142,143]

**Table 4:** Non-invasive imaging techniques to screen for head and neck cancer (HNC) have mainly been applied in vivo so far for oral cancer.

Imaging Technique	Comment
Tissue autofluorescence	Sensitivity of 81% but very low specificity of 50%, cannot be recommended [146,147]
Tissue reflectance	Via direct illumination with low-wavelength light; pooled sensitivity of 94% but very low specificity of 19–69%; hence, it cannot be recommended [139,147]
Narrow-band imaging (NBI)	Tool for identifying malignant transformation of oral potentially malignant disorders and oral cancer; intra-epithelial papillary capillary loop (IPCL) classification II or above is recommended to undergo biopsy; pooled sensitivity of 87–96% and specificity of 83–98%. Medical devices available [148,149,150,151,152]
Optical coherence tomography (OCT)	Also, a tool for the detection of oral cancer; pooled sensitivity of about 91% and specificity also of 91% [153]; implementation of machine learning algorithms might help to improve the accuracy [154]
Contact endoscopy	Analyzed for use in the oral cavity, pharynx, and larynx. Can reach a sensitivity and specificity of >95%. Needs experience in the interpretation of the vascular structure [155,156,157,158]. Contact endoscopy. Medical devices available
Confocal endomicroscopy	Confocal laser endomicroscopy combined with the contrast agents acriflavine or fluorescein and machine learning. Accuracy in two studies was sensitivity 81–86.8%, specificity 92–95%. Medical devices available [158,159]
Reflectance spectroscopy	This was used to field cancerization in the oral cavity in patients with laryngeal cancer [160], but not for screening for oral cancer; as polarized reflectance spectroscopy with a sensitivity 74–80%, specificity 80–93% [161]; might be used in the future also for screening when improved by the use of deep learning analysis [162]
Elastic scattering spectroscopy	Just used in a few studies; sensitivity of 72% and a specificity of 75% [163]
Raman spectroscopy, including surface-enhanced Raman spectroscopy (SERS) and shifted-excitation Raman difference spectroscopy (SERDS)	When coupled with deep learning, both sensitivity and specificity can reach values > 95% [164]

## Data Availability

The original contributions presented in the study are included in the article; further inquiries can be directed to the corresponding author.
